# Supplementary material for: Geographical Distribution, Host Range and Genetic Diversity of Fusarium oxysporum f. sp. cubense Causing Fusarium Wilt of Banana in India
Source: J Fungi (Basel). 2024 Dec 21;10(12):887. doi: 10.3390/jof10120887 (PMC11679753; doi:10.3390/jof10120887)
Supplement: Supplementary file 1 [file jof-10-00887-s001.zip › jof-3339162-supplementary.pdf]

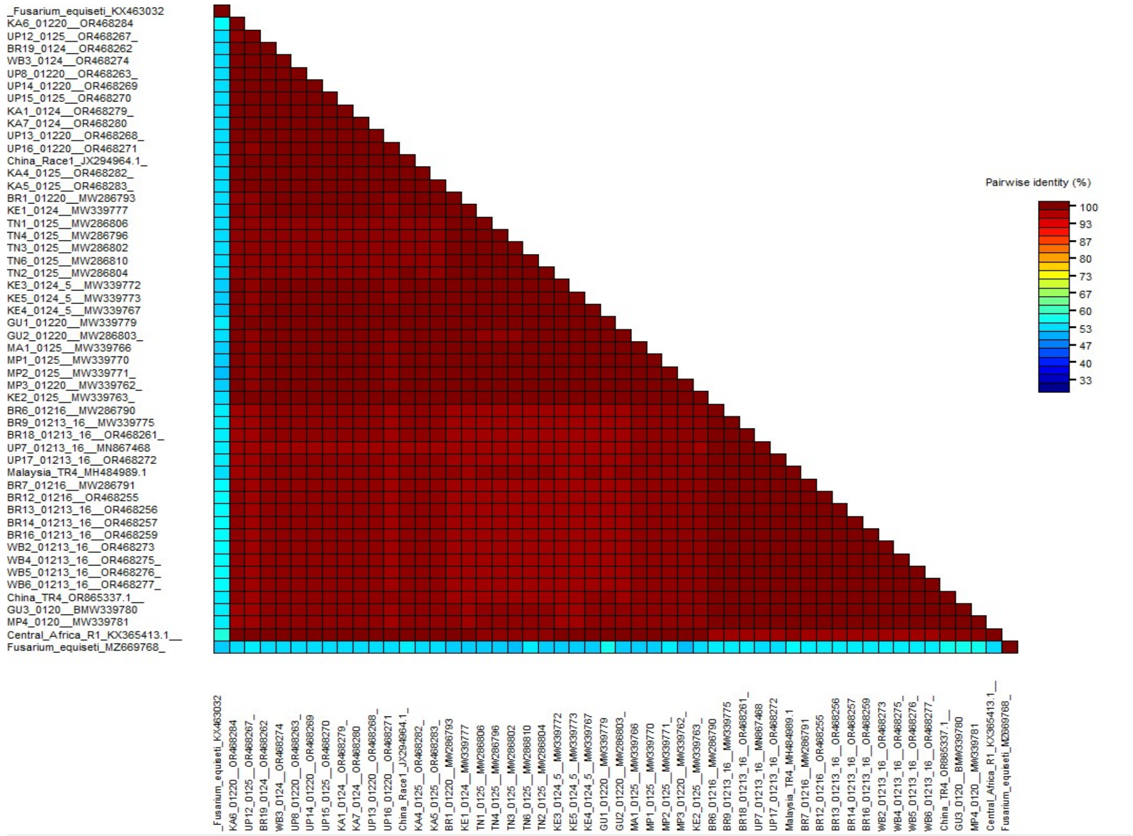

Supplementary Figure S1. Similarity index was assessed by using Sequence Demarcation Tool (Version 1.3)
